# The Pediatric Crohn Disease Morbidity Index (PCD-MI): Development of a Tool to Assess Long-Term Disease Burden Using a Data-Driven Approach

**DOI:** 10.1097/MPG.0000000000003793

**Published:** 2023-04-20

**Authors:** James J. Ashton, Abhilasha Gurung, Cai Davis, Eleanor G. Seaby, Tracy Coelho, Akshay Batra, Nadeem A. Afzal, Sarah Ennis, R. Mark Beattie

**Affiliations:** From the * Department of Human Genetics and Genomic Medicine, University of Southampton, Southampton, UK; the † Department of Paediatric Gastroenterology, Southampton Children’s Hospital, Southampton, UK; ‡ NIHR Southampton Biomedical Research Centre, University Hospital Southampton, Southampton, UK.

**Keywords:** Crohn disease, pediatric, prediction

## Abstract

Heterogeneity and chronicity of Crohn disease (CD) make prediction of outcomes difficult. To date, no longitudinal measure can quantify burden over a patient’s disease course, preventing assessment and integration into predictive modeling. Here, we aimed to demonstrate the feasibility of constructing a data driven, longitudinal disease burden score.

**Methods:**

Literature was reviewed for tools used in assessment of CD activity. Themes were identified to construct a pediatric CD morbidity index (PCD-MI). Scores were assigned to variables. Data were extracted automatically from the electronic patient records at Southampton Children’s Hospital, diagnosed from 2012 to 2019 (inclusive). PCD-MI scores were calculated, adjusted for duration of follow up and assessed for variation (ANOVA) and distribution (Kolmogorov-Smirnov).

**Results:**

Nineteen clinical/biological features across five themes were included in the PCD-MI including blood/fecal/radiological/endoscopic results, medication usage, surgery, growth parameters, and extraintestinal manifestations. Maximal score was 100 after accounting for follow-up duration. PCD-MI was assessed in 66 patients, mean age 12.5 years. Following quality filtering, 9528 blood/fecal test results and 1309 growth measures were included. Mean PCD-MI score was 14.95 (range 2.2–32.5); data were normally distributed (*P* = 0.2) with 25% of patients having a PCD-MI < 10. There was no difference in the mean PCD-MI when split by year of diagnosis, *F*-statistic 1.625, *P* = 0.147.

**Conclusions:**

PCD-MI is a calculatable measure for a cohort of patients diagnosed over an 8-year period, integrating a wide-range of data with potential to determine high or low disease burden. Future iterations of the PCD-MI require refinement of included features, optimized scores, and validation on external cohorts.

## Graphical abstract

**Figure:**
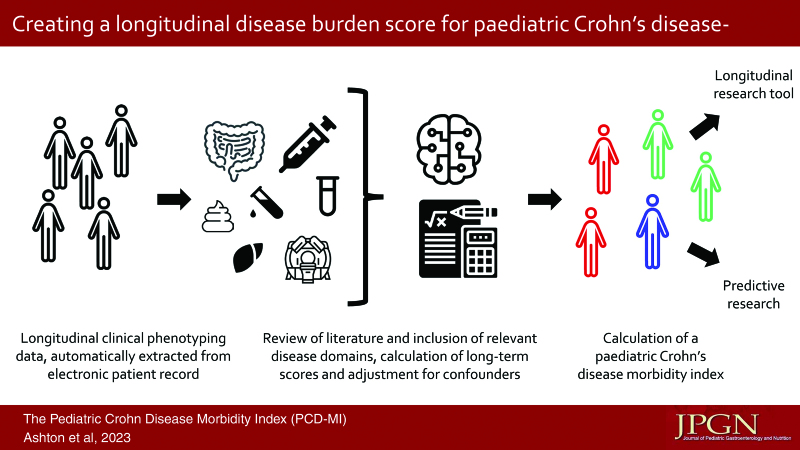

**What Is Known**

- Disease burden is difficult to quantify in Crohn disease (CD), partly due to considerable heterogeneity. Current tools capture single timepoint disease activity rather than longitudinal disease burden.

**What Is New**

- In this study we use a data-driven approach to create the first iteration of a pragmatic longitudinal disease burden score, the pediatric CD morbidity index (PCD-MI).
- We envisage future iterations of the PCD-MI becoming a useful tool for incorporation into disease prediction research, with the next steps to refine the included features, optimize scores, and validate on external cohorts

Crohn disease (CD) is a relapsing and remitting, lifelong condition primarily affecting the intestine. It is a highly heterogeneous condition and the clinical disease course is hugely variable between patients. Pediatric onset patients will have disease for their entire adult lives, with significant variation in disease burden. Many effective therapies exist, however targeted therapy and prediction of outcomes remains mostly theoretical. To date, trials and prospective predictive models have focused on single timepoint outcomes, commonly steroid-free remission at 52 weeks, the need to step-up therapy or occurrence of complicated disease during follow-up (1–4). Recently, there have been renewed calls to prioritize longitudinal assessment of disease to facilitate and promote precision medicine for patients with inflammatory bowel disease (IBD) (5).

Real world data has the potential to guide predictive models, including application of artificial intelligence algorithms to predict long-term outcomes and enable optimization of treatment with a realistic and evidenced discussion of potential risks (6). Single time point disease activity scores are well established as clinical and research tools, both to define remission and to guide escalation of therapy (7). However, these only reflect a snapshot of disease burden. Longitudinal cohort studies aiming to determine predictors of disease activity are currently restricted to pragmatic proxies of long-term disease burden, with longitudinal disease burden scores currently lacking for CD. Well-defined and reproducible long-term assessment of disease burden has the potential to improve patient stratification and aid with discovery of clinical and molecular predictors of disease (8). Recently it has been suggested that long-term disease activity and severity assessment should include three domains: impact of disease on a patient, measurable inflammatory burden, and disease course (including complications) (9).

There is no current benchmark for the assessment of longitudinal disease. In this study we aimed to demonstrate what is feasible with a data-driven approach to creating a pragmatic longitudinal disease burden score. We term this developmental tool, the pediatric CD morbidity index (PCD-MI), and include evidence from current activity scores and contemporary guidelines. We provide preliminary testing of the PCD-MI on a cohort of pediatric patients with variable follow-up times and assessed the ability of the tool to discriminate between high and low disease burden.

## METHODS

### Review of Existing Evidence

Tools utilized for assessing disease activity in pediatric and adult-onset CD were retrieved through structured review of the literature using the search strategy: (Crohn’s disease) AND (score) OR (tool) OR (index) AND (activity) in the Medline database (accessed July 26, 2022).

Clinical parameters from these tools assessing objective long-term measures of disease activity were extracted and utilized to develop the disease burden tool. Consensus European Crohn’s and Colitis Organisation-European Society for Paediatric Gastroenterology, Hepatology and Nutrition (ECCO-ESPGHAN) pediatric CD guidelines were reviewed for treatment and management strategies reflecting differing disease courses (10). Long-term treatments and measures used to monitor response were retrieved and incorporated into the disease burden assessment tool.

### Clinical Data Extraction

Patients were identified from Southampton Children’s hospital (SCH) pediatric IBD database. The study included patients from January 1, 2012 to December 31, 2019. SCH cares for patients aged <18 years with IBD referred from 12 district general hospitals in the South of England. To be included, patients had to have a diagnosis of CD, live locally to Southampton, and have a Southampton (SO) postcode (to ensure blood and stool monitoring were performed at SCH rather than in a referring/shared care unit), and have a minimum follow-up time of 2 years.

Clinical data were automatically extracted from electronic health records (EHRs) through partnership with the University Hospital Southampton Digital and Southampton Biomedical Research Centre Data Science. Duration of follow-up from diagnosis until last clinical contact was calculated for all patients. Based on features implicated through review of the existing evidence the following results were retrieved from the EHR.

#### Longitudinal Blood and Stool Results

All results underwent quality control measures. Measures that were biologically implausible, reported an insufficient sample, failed test or sample not received, or that were hemolyzed were excluded from downstream analyses as previously described (11). All measures were retrieved with age- and sex-specific normal ranges.

Measures, including C-reactive protein, albumin, hemoglobin, platelets, and fecal calprotectin were individually plotted overtime using the ggplot2 package in “RStudio” (build 492). For each measure, patients were extracted and plotted, and those with less than 5 data points for that measure were excluded. Using the abnormality flags for each test result (based on each test’s normal range), individual patients were classified into three disease activity groups for each measure: (1) Initial abnormal (and normalizes by 1 year), (2) Persistently normal (including at diagnosis), and (3) Relapsing and remitting abnormality (over disease course). No patients had <1 year of follow-up. We would recommend that any future inclusion of patients with <1 year of blood/fecal results should be assigned to group 1 if their results had normalized, or group 3 if their results showed ongoing inflammation.

Patients were annotated with their disease activity group and each group was plotted using ggplot2 to illustrate the trajectory of results over time. This was conducted for each blood/stool measure. Blood results included from our cohort represent the data available for our cohort; as results are included as longitudinal patterns to determine disease trajectory it would be possible to calculate a score using additional or alternative blood results (such as erythrocyte sedimentation rate) that could be tailored for a cohort, so long as these results represented inflammation or disease activity.

#### Disease Behaviors

Endoscopy, small bowel magnetic resonance imaging (MRI), abdominal ultrasound, clinic letters, and CT abdomen scan reports were retrieved from the University Hospital Southampton EHR. These records, including clinic letters, imaging reports, and endoscopy reports, were electronically searched for stricturing (Fibrosis, Fibrotic, Stricture, Stricturing, Narrowing, Narrowed, Pre-stenotic dilatation, Stenotic, Reduced diameter) and fistulating (fistulating, fistula, fistulae, fistulising, fistulizing, penetrating, penetrate, penetrative, connection, connect, connecting) disease keywords to reduce the number of reports requiring clinical curation. Records without key words were recorded as a not-stricturing or non-penetrating phenotype, and the remaining were manually checked by a clinician to assign patients as having the correct disease behavior.

#### Medications and Surgery

Electronically held clinic letter and infusion records were manually checked for each patient to determine which long-term medications they had been prescribed during the follow-up period. Long-term medication was coded as a binary outcome for individuals and does not reflect starting or stopping therapies.

Surgical procedures were extracted from the EHR. All operation notes are stored in a standardized format. The name of the operation was retrieved and classified by a clinician (JJA) as an intestinal resection, perianal procedure (including seton placement, abscess drainage but excluding examination under anesthesia), or an operation unrelated to CD.

#### Growth Measures

Weight and height standard deviation scores (SDS) were retrieved from the EHR from diagnosis to the most recent follow-up time. SDS underwent quality control as previously described (12). Change in SDS from diagnosis to most recent measure was calculated. Patients with an SDS at most recent follow-up <2.0 were flagged.

#### Extraintestinal Manifestations (EIMs)

Clinic letters and discharge summaries from the EHR were manually checked for each patient to determine doctor-diagnosed EIM of CD and concurrent autoimmune disease. Specifically, the presence of liver (concurrent autoimmune hepatitis, autoimmune sclerosing cholangitis, and primary sclerosing cholangitis), skin (pyoderma gangrenosum, erythema nodosum), eye (uveitis, iritis), and arthritis (peripheral and axial) were assessed.

### Calculation of PCD-MI

Extracted data were summarized for each patient. Based on longitudinal outcome domains identified from previous activity indexes and published guidance, scores were assigned to outcomes within each of the domains. The relative importance of clinical factors within the score was reflected in the assigned value for that clinical variable. These data were derived from previous activity index scores which attributed values to clinical parameters, where available.

### Modification of Scores Based on Follow-Up Duration

Based on the presumption that patients with longer disease duration are more likely to develop complications, escalation of therapy, occurrence of surgery, and EIMs (13–15), a follow-up duration modification coefficient was calculated to upweight high disease burden occurring within shorter follow-up times. The mean PCD-MI score was calculated, per year of follow-up and this value was divided by the mean overall PCD-MI score. Scores were banded by follow-up duration and the factor needed to normalize the PCD-MI score for that follow-up duration was calculated. This value was used as the follow-up duration modification coefficient. As no patients with <1 year of follow-up were included the coefficient for this group was extrapolated from the included data (Table 1, Supplemental Digital Content, http://links.lww.com/MPG/D130).

### Assessment of the PCD-MI

Through inclusion of additional features in the PCD-MI such as long-term inflammation (blood results, fecal calprotectin), complications, and the need for surgery, we aimed to make scores from different standard clinical practice timepoints comparable. To assess this, we compared the scores of patients diagnosed in each year of study, accounting for variable follow-up time using the follow-up coefficient, and assessed for differences using ANOVA.

### Ethics

This study was deemed to be a service evaluation and registered at University Hospital Southampton.

## RESULTS

### Construction of PCD-MI

#### Tools Which Assess Disease Burden

The pediatric CD activity index (PCDAI) score, and modifications of the PCDAI, wPCDAI, shPCDAI, abbrPCDAI, the Harvey-Bradshaw index (HBI), and the CD activity index (CDAI) were identified from the literature (7,16–18). From these tools features that would be applicable over time were extracted (Table 1). Measures reflecting transient proxies of disease activity, such as number of stools per day, abdominal pain on a specific day, and general wellbeing on a specific day, were excluded as they do not reflect long-term disease burden (9). Tools and scores requiring contemporaneous interpretation of endoscopic or radiological data were not included.

**TABLE 1.** Summary of features common to the 3 major disease activity score indices: Pediatric Crohn disease activity index, Crohn disease activity index (adult tool), and the Harvey-Bradshaw index (adult tool), alongside the latest ECCO ESPGHAN pediatric Crohn disease guidelines

	Pediatric Crohn disease activity index	Crohn disease activity index (adult tool)	Harvey-Bradshaw index (adult tool)	ECCO-ESPGHAN 2020 guidelines
Medication and treatments	None	Antidiarrheal drugs	None	Treatment algorithms with stepwise escalation through immunomodulation, biologics, and additional therapy such as surgery
Disease complications—fistulating, stricturing, or abdominal masses	Includes fistulating and abdominal masses	Includes fistulating and abdominal masses	Includes fistulating and abdominal masses	Risk stratification of patient based on disease behavior—B2 and B3 disease = “high risk”
Extraintestinal manifestations	Yes—arthritis, uveitis, erythema nodosum, or pyoderma gangrenosum	Yes—arthritis or arthralgias, iritis or uveitis, erythema nodosum, pyoderma gangrenosum	Yes—arthralgia, uveitis, erythema nodosum, pyoderma gangrenosum	Not mentioned
Investigation results	Yes—full blood count related measures, inflammatory markers, and albumin	Yes—full blood count related measures	None	Monitoring using fecal calprotectin and prospectively collected endoscopic measures of disease extent/severity—Simple Endoscopic Score for Crohn Disease (SES-CD), or Crohn Disease Endoscopic Index of Severity (CDEIS)
Growth measures	Height and weight assessment	Assesses weight loss	None	Growth delay is associated with severe disease and “medium risk” stratification

#### Themes Assessing Long-Term Outcomes

Features assessing longitudinal clinical features from each tool (PCDAI, HBI, and CDAI) were classified by theme: “medication and treatments,” “disease complications,” “extraintestinal manifestations,” “investigation results,” and “growth measures” (Table 1).

All themes were then cross-referenced with the ECCO-ESPGHAN guidelines to include additional data pertinent to long-term disease burden; this included specific information on treatment algorithms, disease behavior, investigations, and growth. This resulted in a list of disease burden domains to include in the PCD-MI which included blood results, fecal calprotectin results, medication, complications, surgery, growth, and EIMs (10).

#### Features Included in the PCD-MI

Clinical data from disease burden domain were used to populate the PCD-MI. Features within domains were assigned values reflecting proxies of long-term disease burden. These values were then scaled to reflect an overall score with a minimal value of 0 and a maximal value of 50 (Table 2). It is possible to achieve a score above 50 if more than 4 EIMs of IBD are present.

**TABLE 2.** Clinical variables included in the pediatric Crohn disease morbidity index (PCD-MI) with the accompanying score

Disease burden domain	Components	Score
Longitudinal blood results	Hemoglobin group 1, 2 + 3*	0, 1, 3
	C-reactive protein group 1, 2 + 3*	0, 1, 3
	Platelets group 1, 2 + 3*	0, 1, 3
	Albumin group 1, 2 + 3*	0, 1, 3
Longitudinal calprotectin	Fecal calprotectin group 1, 2 + 3*	0, 1, 3
Medication	5-ASA/topical treatment	1
	Immunomodulator	2
	First line monoclonal	3
	Second line monoclonal or small molecule	4
Complications	Fistulating disease	3
	Stricturing disease	3
Surgery	Intestinal resection (ever)	4
	Perianal procedure (ever)	2
Growth	Fall of >1.0 weight SDS from baseline, dropping to below −2.0 SDS during follow-up or no improvement from SDS < −2.0 at baseline	2
	Fall of >1.0 height SDS from baseline, dropping to below −2.0 SDS during follow-up or no improvement from SDS < −2.0 at baseline	2
Extraintestinal manifestation	Autoimmune liver disease—primary sclerosing cholangitis, autoimmune sclerosing cholangitis, autoimmune hepatitis	3
	Chronic skin disease—psoriasis, pyoderma gangrenosum, etc	2
	Autoimmune eye disease—uveitis, episcleritis, etc	2
	Arthritis—peripheral or axial	2
	And/or other autoimmune comorbidity related to IBD	−2
		Min = 0
		Max = 50 (+)

* Group 1 = persistently normal result, group 2 = initially abnormal and normalizes over disease course, group 3 = relapsing and remitting abnormality over disease course.

IBD = inflammatory bowel disease; SDS = standard deviation score.

For longitudinal data (blood results, fecal calprotectin) each patient’s data were modeled over their follow-up duration and classified into 1 of 3 groups, as described above: (1) Initial abnormal (and normalizes), (2) Persistently normal (including at diagnosis), and (3) Relapsing and remitting abnormality (over disease course).

Growth measures were converted to SDS. For growth we defined a significant disease burden as a fall in weight or height of >1.0 SDS from baseline, or no improvement from SDS < −2.0 at baseline. This allowed the score to account for patients whose natural growth potential was lower, while identifying patients lying outside 2 standard deviations or with long-term growth deficits following diagnosis.

Use of specific medications, occurrence of complicated disease (stricturing or fistulating disease), occurrence of surgery, and EIMs were included as binary outcomes, that is, if they had occurred for a patient, they were scored for that patient.

#### Modification of Scores Based on Follow-Up Duration

We implemented a follow-up duration modification to the basic PCD-MI score as described. Follow-up duration was based on normalized mean PCD-MI for each year of follow-up. Comparing this value to the mean PCD-MI score for the whole cohort allowed a per year normalization factor to be calculated. After normalizing this to 10 years of follow-up we then banded the years of follow-up together to give the following coefficients: <1 year (PCD-MI score × 2), 1–3 years (PCD-MI score × 1.5), 5–10 years (PCD-MI score × 1.1), and >10 years (PCD-MI score × 1) (Table 1, Supplemental Digital Content, http://links.lww.com/MPG/D130).

### Calculation of PCD-MI

We calculated the PCD-MI scores for a cohort of patients with automatically extracted follow-up data.

#### Testing Cohort-Patient Identification and Data Extraction

**FIGURE 1.**
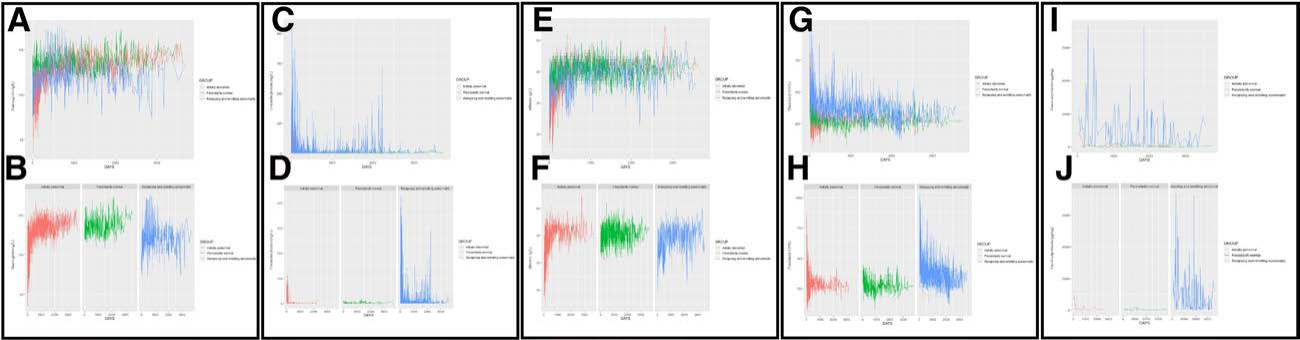
Blood and calprotectin results. Patients included in pediatric Crohn Disease morbidity index (PCD-MI) validation plotted over the duration of follow-up, shown as collated results and individual graphs for separate patient groups: initially abnormal (red), persistently normal (green), and relapsing and remitting abnormality (blue). (A) Hemoglobin collated results, (B) hemoglobin by patient group, (C) C-reactive protein collated results, (D) C-reactive protein by patient group, (E) albumin collated results, (F) albumin by patient group, (G) platelets collated results, (H) platelets by patient group, (I) fecal calprotectin collated results, and (J) fecal calprotectin by patient group.

All patients diagnosed between January 1, 2012 and December 31, 2019 and living within Southampton were included. Seventy patients had available data. Following exclusion of patients with less than 5 blood result data points across their follow-up period, a total of 66 patients were included in the testing cohort, mean age 12.5 years, 24 female (36.4%). Following exclusion of physiologically implausible values, hemolyzed, and clotted samples this data set included 2078 CRP measures, 2437 hemoglobin measures, 2434 platelet measures, and 2367 albumin measures. Each patient was assigned to 1 of the 3 longitudinal groups (groups 1, 2, and 3) for each blood result measurement (Fig. 1A–H).

There were 212 fecal calprotectin measures available. All patients had at least 1 fecal calprotectin result available and were assigned to 1 of the 3 longitudinal groups. As expected, all patients were assigned to normalization after diagnosis or persistent relapsing and remitting abnormality, with no patients having a normal calprotectin at diagnosis. There was significant sparsity of data for fecal calprotectin, reflecting the initial diagnosis for some patients being >10 years ago. Despite this, any abnormal results since diagnosis allowed classification into the persistent relapsing and remitting abnormality group, with all other patients falling into the normalized or normal throughout groups (Fig. 1I and J).

#### Growth Measures

A total of 1309 individual growth measures were recorded, consisting of 604 height measures and 705 weight measures. Following exclusion of physiologically implausible values, 601 height measures and 702 weight measures were included in the analysis.

No patients had a SDS < −2.0 at diagnosis and showed no growth improvement, 3 patients had a height SDS < −2.0, and 10 patients had a weight SDS score of <−2.0. Three patients dropped height SDS by >1.0 SDS during follow-up, but no patients dropped weight by >1.0 SDS (Table 1, Supplemental Digital Content, http://links.lww.com/MPG/D130).

#### PCD-MI Calculation for Testing Cohort

**FIGURE 2.**
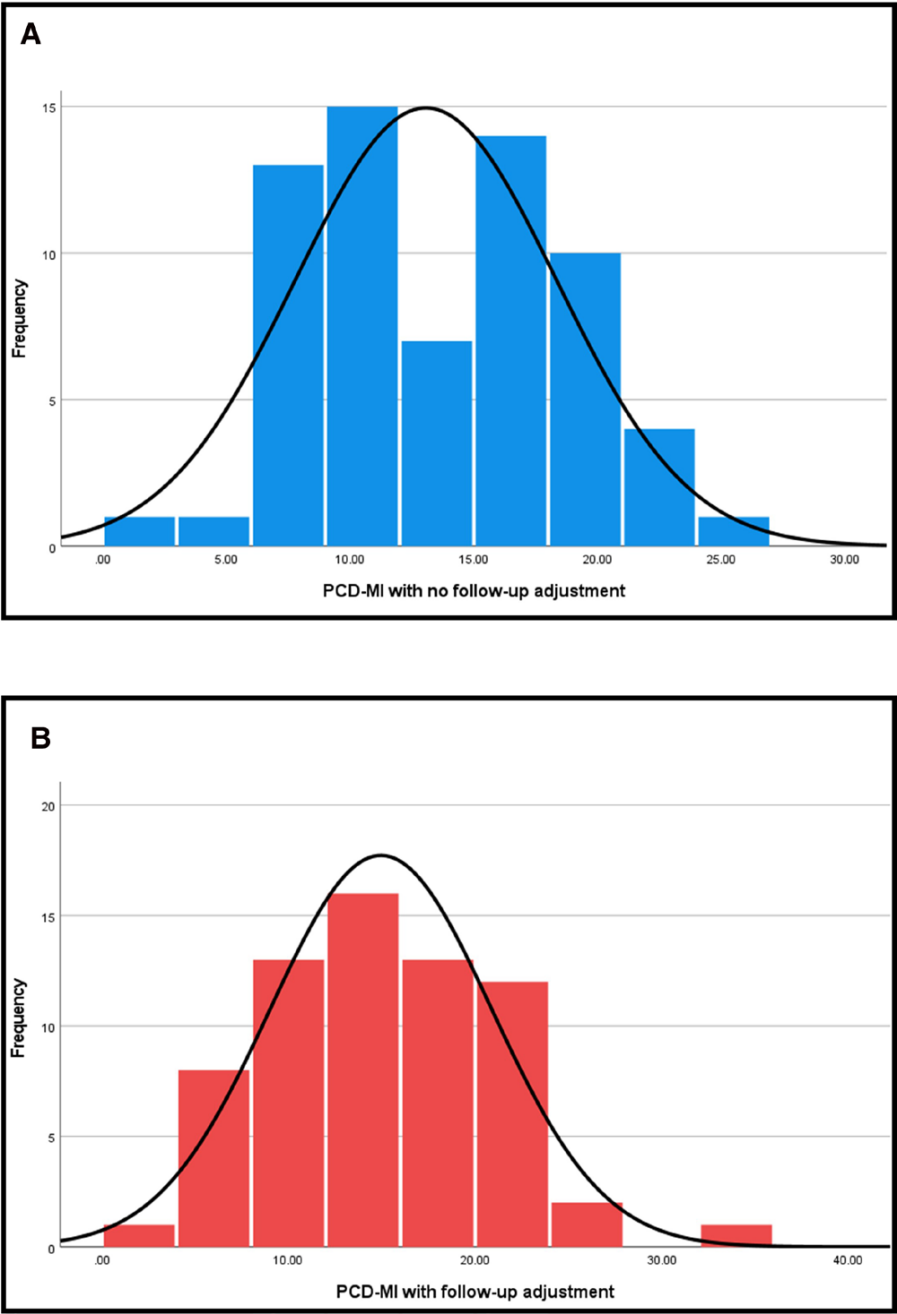
Histograms of patients included in pediatric Crohn Disease morbidity index (PCD-MI). Validation, bin size 5 points. (A) Distribution of patients prior to application of follow-up coefficient and (B) distribution of patients after the application of follow-up coefficient.

Using the values assigned to each clinical data measure we calculated the PCD-MI score for each patient. The mean PCD-MI score prior to application of the follow-up coefficient was 13.06. After application the mean PCD-MI score was 14.95. Scores were then plotted on a histogram to demonstrate the distribution within this cohort (Fig. 2A). The distribution of the PCD-MI without follow-up coefficient data was normal according to the Kolmogorov-Smirnov statistic 0.106, *P* = 0.062. Each score was then multiplied by the follow-up coefficient for that patient, and replotted as a histogram (Fig. 2B). The distribution of the PCD-MI with follow-up coefficient data was normal according to the Kolmogorov-Smirnov statistic 0.069, *P* = 0.2.

#### Assessment of the Tool

Patients were split by year of diagnosis and the mean PCD-MI score, with follow-up coefficient, calculated for each year (Table 1, Supplemental Digital Content, http://links.lww.com/MPG/D130). Following analysis with ANOVA, no significant differences were shown between the year groups, demonstrating comparability of PCD-MI scores across the whole cohort, regardless of year of diagnosis.

The highest total possible PCD-MI score for our test cohort was 75, as the minimum follow-up time for an individual was 2.75 years. The highest score we observed was 32.5, with the lowest 2.2. A total of 17 patients (25.8%) had a PCD-MI score of ≤10, mean follow-up 6.2 years (range 2.7–10.5 years), demonstrating long-term quiescent disease. Similarly, 15 patients (22.7%) had scores ≥20, mean follow-up 5.9 years (range 2.9–9.7 years), demonstrating a higher burden of longitudinal disease. Importantly for a representative score across a cohort, most patients (51.5%) fell between a PCD-MI score of 10 and 20, reflected by the normal distribution of the scores.

## DISCUSSION

Using a data-driven approach and incorporating key longitudinal disease variables from previously validated indices and guidelines we have created the first iteration of a disease burden tool for pediatric CD, capable of assigning a long-term score based on readily available clinical data. This score provides a potential mechanism for prediction of long-term disease outcomes for incorporation into disease prediction models, and represents a staging post in the development of a collaborative and evidence-driven burden score. Further development, rationalization, and testing will provide the potential for the PCD-MI to compare patients with different follow-up points and treated at different timepoints, as it utilizes numerous clinical data inputs, reflecting conventional practice continuing to change over time. For an individual patient it would also be possible to assess PCD-MI over time through calculation at intervals. To our knowledge no other composite longitudinal scores of disease burden exist for clinical or research purposes, and this study presents a demonstration of what is feasible in the era of “big clinical data.” It is not intended to replace current, validated, disease scores, such as PCDAI, that are used to assess single timepoint disease activity, and is intended as a first step towards a consensus disease burden tool.

Given the numerous different elements of the score, and the heterogeneity of CD, not many patients were able to achieve very high scores as different phenotypic subgroups are less likely to have overlapping features with other groups, for example penetrating and stricturing disease (13). This is a comparative strength of the PCD-MI, allowing patients to achieve scores for disease burden from a variety of different disease processes. It also highlights that a number of patients, 25% in this cohort, have long-term quiescent disease which is similar to previously reported by Wintjens *et al* (19), who described 28.2% of adult patients being classified to a long-term quiescent cluster. Identifying these patients at the point of diagnosis would present huge potential to avoid significant therapy, minimize side effects and costs, while also not exposing patients to long-term risk of developing disease complications.

The need for long-term disease assessment have been highlighted by a number of consortia focused on precision medicine in IBD (20,21). Where previous prediction studies in CD have used pragmatic proxies of long-term disease activity, such as the need to step up therapy, or specific disease complications, there is a clear requirement to more accurately phenotype longitudinal disease (1,2). An example of this is reflected by a number of studies utilizing surgery as a proxy for severe disease (22,23). While occurrence of surgery is clearly an important factor in disease burden, for some patients with CD an isolated right hemicolectomy may occur after a long period of quiescent disease and result in further long-term well-controlled disease. The PCD-MI allows reflection of the total disease burden, rather than relying on a single measure of disease activity over time.

Similar to our study, previous efforts have been made to classify long-term disease activity. Cosnes *et al* (24) describe a consensus method for discriminating severe from a mild-to-moderate CD course in adult patients; however this study did not utilize a data-driven approach and relied on expert opinion. More recently Chen *et al* (25) have described a longitudinal cluster analysis of patient’s blood results to determine different clusters of disease activity related to infliximab treatment, reflecting a data-driven approach but limited only to blood results. Jiang *et al* (26) incorporated health care costs and disease metrics, including tracking HBI over time to determine subgroups of disease trajectories. The methodology and data used to derive these differing longitudinal scores is largely reflected in our PCD-MI, with the added benefit that we are able to incorporate large amounts of clinical data collected over the follow-up of a patient.

The main utility for the PCD-MI may be as a numerical score for incorporation into research, to predict long-term disease activity. Scores are likely to mean little if applied in isolation to a patient but are more useful when observed across a cohort. With the vast amount of data now being generated through initiatives such as the United Kingdom IBD BioResource and the International IBD Genetics Consortium, there is a need to improve the precision and usefulness of clinical data collected alongside these projects (27,28). Without accurate clinical data, and disease burden metrics, to incorporate into prediction models, the clinical translation of these resources will remain limited. The largest potential may be for patients with long-term quiescent disease, where therapy can be minimized, prevented associated risks, reducing medication burden and costs, without the risks of disease complications or flares. We also point to the ability to include additional longitudinal blood or fecal results that are used to assess disease activity in a specific cohort or hospital, such as erythrocyte sedimentation rate, into an iteration of PCD-MI that can then be used to compare disease burden longitudinally within a local cohort.

The study and PCD-MI score have strengths, but we also acknowledge potential weaknesses. A potential criticism of this score is that the values assigned to each clinical variable that comprise the score are not always evidence based. We accept that these values are often pragmatic opinion;, however, this strategy also reflects the initial PCDAI development (18). In terms of blood and fecal pathology results, we recognize that retrospectively gathered patient data will frequently have missing data points, but it may be possible to interpolate results in some circumstances. However, through a longitudinal classification of the pattern of patient results there is also the ability to compensate for this. Another potential strength is the flexibility the score offers when including blood results. Different cohorts will have differing blood results available and the score could accommodate different tests or values that reflect disease activity or inflammation. We have also focused on pediatric-onset patients; however, with minor adjustments this score could be utilized as an adult CD morbidity index. The future inclusion of standardized measures of disease extent through endoscopic, histological, and radiological tools would also be desirable (29,30). To date, the retrospective scoring of these tools and the lack of standardization of repeating radiological or endoscopic investigations was too limited to include in this iteration; however, as electronic data capture improves then we would envisage these tools being incorporated into PCD-MI. It is also not possible to have retrospective measures of the impact of disease on a patient, which would be an important prospective modifier for the score. Some of the calculations require manual steps, requiring additional time to calculate the score. We acknowledge the rate of EIMs in this cohort is approximately 8%, lower than the estimated 20% generally reported in the literature (31). Despite this, our data are restricted to arthritis, liver disease, oral and dermatological manifestations, or another autoimmune complication of IBD, and we have not included aphthous stomatitis/ulcers, which account for a large proportion of the described EIM in the literature (31). The PCD-MI will require validation on external cohorts to assess its ability to translate across health care systems; however, with the increasing use of electronic health care records the ability to calculate this score will become easier. Further modifications and refinements of the PCD-MI will be useful over time, including refinement of the data included, the weighting of individual clinical variables, and the calculation of follow-up coefficient. The result of the score is a continuous variable related to disease burden, rather than a categorical high/low burden. For the purposes of integration into subsequent data analyses we believe a numerical value is more useful. Validation on external cohorts, and expert opinion, could result in a future consensus on values deemed to reflect high/low disease burden.

## CONCLUSIONS

This study uses an evidence and data-driven approach to demonstrate the feasibility of a longitudinal PCD-MI. This score was calculated on a cohort of patients diagnosed over an 8-year period and assessed for variation between years of diagnosis and number of years of follow-up. We envisage future iterations of the PCD-MI becoming a useful tool for incorporation into disease prediction research, with the next steps to refine the included features, optimize scores, and validate on external cohorts.

**Table:** 

Follow-up time modification	Multiplicative factor
Score calculated <1 year	×2.0
Score calculated 1–3 years	×1.5
Score calculated 3–5 years	×1.25
Score calculated 5–10 years	×1.1
Score calculated >10 years	×1.0

Follow-up coefficients related to duration of time since diagnosis for which an individual patient has follow-up clinical data for, Table 1, Supplemental Digital Content, http://links.lww.com/MPG/D130.

## Supplementary Material


