# Supplementary material for: The Pediatric Crohn Disease Morbidity Index (PCD-MI): Development of a Tool to Assess Long-Term Disease Burden Using a Data-Driven Approach
Source: J Pediatr Gastroenterol Nutr. 2023 Apr 20;77(1):70–8. doi: 10.1097/MPG.0000000000003793 (PMC10259218; doi:10.1097/MPG.0000000000003793)
Supplement: Supplementary file 1 [file mpg-77-70-s001.pdf]

Year of diagnosis	Number of patients	Mean PCD-MI	Std. Deviation	Std. Error	95% Confidence Interval for Mean	
					Lower Bound	Upper Bound
2012	12	14.9333	4.68000	1.35100	11.9598	17.9069
2013	7	15.8714	4.61726	1.74516	11.6012	20.1417
2014	8	10.8625	4.60867	1.62941	7.0096	14.7154
2015	4	17.8750	6.86215	3.43108	6.9558	28.7942
2016	6	20.3500	2.93104	1.19659	17.2741	23.4259
2017	8	13.3063	5.72060	2.02254	8.5237	18.0888
2018	10	15.2500	7.83511	2.47768	9.6451	20.8549
2019	11	14.2727	6.60157	1.99045	9.8377	18.7077
Total	66	14.9508	5.94265	.73149	13.4899	16.4116

Supplementary Table 1- *Number of patients included in the PCD-MI validation cohort, included by year of study, with mean PCD-MI (with correction by follow-up coefficient). ANOVA indicates no significant difference between groups, F statistic 1.625, p=0.147*
